# Editorial: Cellular Senescence and Cellular Communications Within Tissue Microenvironments During Aging

**DOI:** 10.3389/fphys.2022.890577

**Published:** 2022-04-11

**Authors:** Ziyu Wei, Heng Ma, Evandro Fei Fang, Hou-Zao Chen

**Affiliations:** ^1^ State Key Laboratory of Medical Molecular Biology, Department of Biochemistry and Molecular Biology, Institute of Basic Medical Sciences, Chinese Academy of Medical Sciences and Peking Union Medical College, Beijing, China.; ^2^ Department of Physiology and Pathophysiology, School of Basic Medicine, Fourth Military Medical University, Xi’an, China; ^3^ Department of Clinical Molecular Biology, University of Oslo and Akershus University Hospital, Lørenskog, Norway; ^4^ The Norwegian Centre on Healthy Ageing (NO-Age), Oslo, Norway

**Keywords:** cellular senescence, aging, microenvironments, cellular communications, senescaging

Aging is expressed as a deterioration and frailty of various tissues, accompanied by dysregulation of the pathways essential for homeostasis, resilience, and maintenance (Aman et al., 2021; Fang et al., 2017; Fang et al., 2016). Under normal physiological conditions, many differing types of cells in tissues interact with each other, constructing a steady microenvironment; for example, parenchymal and accessory cells regulate the normal function of tissues, while accessory cells, such as immune and stromal cells, support the core function of parenchymal cells. The specific components forming the microenvironment and the crosstalk among them are puzzles that remain to be solved. In recent decade, great progress has been made in this field from the perspective of transcriptomics, and researchers have completed the construction of a human single-cell atlas of various organs and tissues (Han et al., 2020). In recent years, many novel single-cell examination strategies have been constructed such as single-cell mass spectrometry (Shrestha, 2020), single-cell immunometabolic profiling (Artyomov and Van den Bossche, 2020), and single-cell-based immune cell research (Mogilenko et al., 2021), allowing further exploration in the field of aging.

Cellular senescence is an irreversible process occurring during the aging of an organism. Cell cycle withdrawal, macromolecular damage, dysregulated metabolism, and senescence-associated secretory phenotype (SASP) are all major phenomena occurring during cellular senescence (Hernandez-Segura et al., 2018). The role of senescent cells in various tissues is intriguing. Cellular senescence not only leads to the dysfunction of the affected cells, but also affects other cells in the microenvironment through SASP (Calcinotto et al., 2019). How senescent cells in different tissues interact with other cells and result in the dysregulation of tissues is an interesting topic; for example, the paracrine effects of senescent cells cause normal surrounding cells to become senescent (Childs et al., 2015). However, which component in different tissues triggers the cellular senescence in various cells and consequently dysregulated tissues is also ambiguous.

In this research topic, we received one original research article and three reviews that focused on the role of cellular senescence and aging-related cellular microenvironments at several levels, including molecular, cellular, and tissue. These works uncovered a relatively comprehensive understanding of aging, and provided future perspectives for research on aging and aging interventions.

At the molecular level, cellular senescence exhibits various failures of biomolecule maintenance, including shortening of telomere length. The article “*Loss of Growth Differentiation Factor 11 Shortens Telomere Length by Downregulating Telomerase Activity*” (Wang et al., 2021) reported novel roles of GDF11 in telomere maintenance and cellular senescence *in vitro*, shedding light on new research directions in the field of rejuvenation and aging.

Aside from cell-autonomous mechanisms, factors within the tissue microenvironment are believed to be able to trigger cellular senescence and consequently aging and age-associated diseases, but what kinds of molecules matter and how they shape the cellular microenvironment need further study. In the review “*Dynamic Aging: Channeled Through Microenvironment*” (Tang et al., 2020), the authors reviewed the role of SASP factors and non-SASP circulating protein factors and metabolites in the aging microenvironment. Various systems and organs were included to illustrate the role of SASP in modulating the development of aging. What’s more, NAD^+^, circulating proteins, and RNA molecules were discussed as circulating factors that play major roles in regulating intercellular communications and shaping the aging microenvironment. Research on aging-related molecules in the circulating and microenvironments is expected to be able to provide novel ideas for the development of senolytic drugs.

Two reviews also summarized the findings related to aging from the perspective of specific organs: brain and bone. In the review entitled “*Beneficial Effects on Brain Micro-Environment by Caloric Restriction in Alleviating Neurodegenerative Diseases and Brain Aging*” (Zhang et al., 2015), authors discussed the factors in the brain microenvironment during aging and aging-related disease, including inflammation, metabolic waste accumulation, damage in permeability of the blood-brain barrier, and epigenetic factors. And in “*Crosstalk Between Senescent Bone Cells and the Bone Tissue Microenvironment Influences Bone Fragility During Chronological Age and in Diabetes*”, (https://www.frontiersin.org/articles/10.3389/fphys.2022.812157/full), authors summarized the factors that promote bone fragility during aging and diabetes, including AGEs, inflammatory RAGE signaling and cell senescence. Interventions based on the therapy of aging such as caloric restriction and clearance of senescent cells are promised to improve health in relevant ageing-related diseases. The most prominent feature of aging is reflected by a large number of seemingly independent diseases in different kinds of tissues and organs. As a result, how it may be possible to target cellular senescence and cellular communications within the tissue microenvironment to prevent these diseases is extremely urgent and warrant further attention.

Almost every cell in the body goes through the process of senescence, and the contribution of senescent cells to the body’s unavoidable aging (we coined this “senescaging”) cannot be ignored. In addition to cellular senescence, more biological events in the microenvironment are included in the aging process of the body (Lopez-Otin et al., 2013; Zhang et al., 2015). Therefore, our current understanding of cellular senescence and cellular communications within tissue microenvironments during aging is still just the tip of the iceberg, and several questions are still outstanding: What is it in tissue microenvironments that triggers cellular senescence and consequently the organism's aging and disease profile? How do senescent cells in different tissues interact with other cells and result in the organism's aging and diseases? What are the underlying mechanisms of cellular senescence and cellular communications within tissue microenvironments, for example in metabolic-, immune-, and endocrine regulation? How can cellular senescence and cellular communications within tissue microenvironments be targeted to prevent age-related diseases, including cardiovascular diseases, cancer, neurological disorders, and other diseases? Nevertheless, increasing attention has been paid to research focusing on the microenvironment (Fane and Weeraratna, 2020; Li et al., 2020; Tang et al., 2020), and more methods are sure to be included in future work, especially single cell sequencing and mass spectrometry, artificial intelligence (Xie et al., 2022), and other new technologies. The development of these strategies will meet the urgent demand for research in the field of senescaging. With joint efforts in the scientific community, the answers to the above questions will become increasingly clear.
